# Probiotics in the Treatment and Prevention of Allergy in Children

**DOI:** 10.1097/WOX.0b013e3181a45ee5

**Published:** 2009-05-15

**Authors:** Erkki Savilahti, Kaarina Kukkonen, Mikael Kuitunen

**Affiliations:** 1 Hospital for Children and Adolescents, Skin and Allergy Hospital, University of Helsinki, Helsinki, Finland

**Keywords:** probiotics, prevention of allergic diseases, treatment of allergic diseases, atopic eczema

## Abstract

Several fold increase in allergic diseases in developed, high-income countries during recent decades is attributed to environmental changes such as urbanization with improved hygiene. This, together with conquering severe bacterial infections during childhood, has reduced the microbial stimulation of the developing immune system of infants. Studies on the pathogenesis of allergy both in man and experimental animal have shown the importance of commensal bacteria in the gastrointestinal tract in stimulating and directing the immune system. The interest in modulating commensal bacterial flora with probiotics to prevent and treat allergy has multiplied in recent years.

In the present review we report results on randomized, controlled studies in which childhood atopic eczema was treated or which aimed to prevent development of allergy during childhood.

Nine studies with 639 patients have looked at the effect of probiotics in treatment of eczema. While 3 studied showed no effect, other studies suggested a moderate benefit of the use of probiotics on the severity of eczema. Studies suggested that the effect may be seen particularly in patients with food allergy and/or sensitization.

Nine studies have reported on the prevention of allergy on 6 study population with altogether 1989 high risk infants. While the early study reporting the development of allergy at ages 2, 4 and 7 years showed a marked reduction of eczema in 77 treated infants, later studies have failed to show similar success. Two studies showed no effect. In the largest study with more than 900 children at age 2 atopic eczema was reduced by 20%, but at age 5 positive effect was present in only the subgroup of children who had born by cesarean section. None of studies has reported adverse effects of probiotics in infants.

Result in both treatment and prevention studies are quite variable, the major reason being the use of different strains of probiotic bacteria and varying types of intervention. Even if the results are encouraging, we need a stronger effect. This may be reached by finding new strains of probiotics affecting stronger stimulation of immune system, together with longer lasting and varying treatment schedules. However, safety issues have to be observed.

## Introduction

The marked increase in the prevalence of allergic diseases during the past 50 years is associated with the decrease of life-threatening infections during childhood and the postindustrial changes in lifestyle, characterized by a high standard of hygiene and reduced exposure to microbes in daily environments [1,2]. Gerrard et al already proposed in the 1970s that this increase is the price we have to pay for better survival of children, although the "hygiene hypothesis" was formulated by Strachan, who found fewer allergies in families with a greater number of children. This led him to postulate that infections protected them from allergy [3,4]. Epidemiologic studies show that persons having had infections of the gastrointestinal tract, like hepatitis A, have lower prevalence of allergies,[5] whereas some other types of infections, for example, measles, may increase the risk for allergy [6].

Other types of stimulation of the mucosal immune system by bacterial antigens, particularly by bacterial endotoxins and lipopolysaccharides, have markedly affected the appearance of allergic diseases during childhood. Increased endotoxin exposure has been associated with living on a farm and results in reduced risk for atopy and for asthma [7,8]. Exposure to unpasteurized farm milk independently (without exposure to the stables) was associated with less atopy and respiratory allergies [9]. Children in families with anthroposophist lifestyle have fewer allergies than other children living in the same area; they use fewer antibiotics, vaccinations are more seldom taken, and they consume fermented foods, plentiful in lactic acid bacteria; only the use of organic or biodynamic fermented foods are significantly associated with the reduction of allergies [10].

The most powerful, direct stimulant of our largest immune organ, gut-associated lymphoid tissue, is the large and active gut microbiota, with up to 100 trillion microbes weighing more than 1 kg. They occupy all available niches from the intestinal lumen to crypts and epithelial-cell surfaces with an increasing gradient from stomach to colon [11,12]. The gut microbiota have huge metabolic activity and through fermentation of their main substrates, undigested dietary carbohydrates, produce short-chain fatty acids (acetate, propionate, and butyrate), carbon dioxide, and molecular hydrogen, salvaging some of the energy of these products for the host [13]. The sterile gut of the newborn is gradually colonized by environmental bacteria. Vaginally born infants acquire the microbiota having the strongest association with the mother's colon [14]. Cesarean section delays fecal colonization by bifidobacteria, lactobacilli, and *Bacteroides*[15,16] and may affect composition of the microbiota up to age 7 [17]. Later, the type of feeding influences the initial colonization [16]. Human milk oligosaccharides promote the growth and activity of bifidobacteria and lactobacilli,[18] which more abundantly colonize breastfed than formula-fed infants [19]. In unhygienic environments, the gut flora has high diversity and a high turnover rate [20]. Such conditions, related to decreased risk for allergies, provide continuous exposure to an extensive array of bacteria in drinking water and in the soil and constantly stimulate the immune system [21].

Several observations indicate that alterations in gut microbiota precede the development of allergies. In 2 countries with either low (Estonia) or high (Sweden) prevalence of allergy, healthy infants had differences in their microbiota [22]. In prospective studies, early fecal samples of infants who go on to develop allergies compared to those who remain healthy grow less enterococci, bifidobacteria, and *Bacteroides *and more clostridia and staphylococci [23]. In a Finnish study of genetically allergy-prone infants, early gut microbiota also differed between those who went on to develop atopy and those nonsensitized by age 1 [24]. In the feces of 5-year-old Esthonian children, those with allergic diseases had less commonly bifidobacteria than those without allergy, whereas clostridia was more common in allergic children. The counts of clostridia correlated with the level of serum IgE in allergic children [25]. Japanese infants developing early allergy had different *Bifidobacterium *species compared to those of nonallergic infants; they particularly had the adult type *Bifidobacterium catenulatum *as has been described earlier in another population [26].

In the experimental animal model for food allergy, the gut microbiota and its stimulatory action of innate immune system by toll-like receptors (TLR), particularly TLR4, is of paramount importance. Food tolerance does not develop in germ-free mice, but is inducible after colonization of the intestine [27]. Mice susceptible to food allergy have a mutation in TLR4, blocking its signaling [28]. The deficiency of TLR4 influenced food sensitization and anaphylaxis to bovine *β*-lactoglobulin depending on the genetic background of the mice [29].

Altering the intestinal microbiota of an individual is a tempting possibility to treat allergic symptoms and to prevent development of allergies. Probiotic bacteria are "live microorganisms, which administered in adequate amounts confer health benefits on the host" (Organization FAO 2002, http://www.who.int/foodsafety/publications/fs_management/probiotics2/en). They are a heterogeneous group of bacteria with specific biological activities. Lactobacilli, bifidobacteria, and streptococci are strains most commonly selected from among human microbiota or dairy-product starters. Lactobacilli, bifidobacteria, and propionibacteria belong to the lactic acid bacteria group. They are fermentative Gram-positive bacteria and produce lactic acid as their main fermentation product. Lactobacilli are naturally present in human and animal intestines and in fermented vegetables or dairy products. *Lactobacillus rhamnosus *GG (LGG), which was isolated from human feces by Gorbach and Goldin, is the most studied probiotic [14]. Bifidobacteria are natural residents of the human intestine, but propionibacteria occur naturally in dairy products and traditionally serve as cheese starters. Prebiotics are indigestible substances that benefit the host by selectively stimulating the growth or the activity or both of a limited number of bacterial strains established in the gut, thereby having impact on allergies. The term "synbiotics" means a combination of these factors.

## Treatment of allergic diseases

Majamaa and Isolauri [30] studied LGG in the treatment of eczema in 42 Finnish infants referred to a hospital for suspected cow's milk allergy in 1997. LGG was given openlabel for 1 month to 11 breast-feeding mothers or randomized directly to 15 infants receiving extensively hydrolyzed formula. In the control group to the latter, 16 infants received only extensively hydrolyzed formula. In the final analysis, 37 of 42 infants undergoing a positive cow's milk challenge after the intervention were included. In these 37, the SCORAD index [31] improved significantly in the 13 formula-fed infants receiving *L. rhamnosus *GG and in the 10 breast-fed infants whose mothers received LGG. In the 14 control infants, the index remained unchanged. However, at 2 months the moderate-to-severe eczema became mild in both study groups. (Table 1). The other Isolauri's study included 27 infants suffering from eczema during exclusive breast-feeding. Of them, 9 were weaned onto extensively hydrolyzed formula, 9 infants onto the same formula with added LGG, and 9 infants received the formula with added *Bifidobacterium lactis *Bb12. After 2 months, in infants receiving the probiotic-containing formulas, severity of eczema decreased significantly, whereas the placebo group showed no improvement; 6 months later, eczema had improved in all infants, with no difference between study groups (Table 1) [32].

**Table 1:** Randomized, Placebo-Controlled Clinical Trials on the Treatment of Eczema With Probiotics

Study	No. Patients	Age	Eczema in Baseline	Sensitized in Baseline	Intervention and Amount of Probiotics (cfu). Duration of Intervention (Weeks)	Clinical Effect
Majamaa and Isolauri (1997)[30]	A1 = 13	2 to 16 months	Moderate to severe, suspected cow's milk allergy	~30%	A1: LGG 5 × 10^8^/g of hydrolyzed formula (HF)	Reduced SCORAD [31]
					C: HF	
	C = 14				Duration: 4 weeks	
Isolauri et al (2000)[32]	A1 = 9	4 to 6 months	Eczema during exclusive breast-feeding	Not given	Infants weaned to:	Reduced SCORAD
					A1: LGG 5 × 10^8^/g in HF	
	A2 = 9				A2: *B. lactis *in HF	
	C = 9				C: HF	
Rosenfeldt et al (2003)[33]	43	1 to 13 years	SCORAD > 15	63%	Double-blind placebo-controlled crossover study:	Reduced subjective symptoms; reduced SCORAD in sensitized children during active period
					A: *L. rhamnosus *10 × 10^10 ^and *L. reuteri *1 × 10^10 ^twice daily	
					P: skimmed milk powder Duration: 6 weeks	
Viljanen et al (2005)[35]	A1 = 80	1 to 12 months	Moderate to severe, referred to hospital for suspected cow's milk allergy	59%	A1: LGG 5 × 10^9 ^and HF	In A1 reduced SCORAD in sensitized infants
	A2 = 76				A2: LGG, *L. rhamnosus *LC705, *Bifidobacterium breve, Propionibacterium freudenreichii *total 1 × 10^9 ^and HF	
	C = 74				C: HF Duration: 4 weeks	
Weston et al (2005)[34]	A = 28	6 to 18 months	Moderate to severe	71%	*L. fermentum *VRI-003 2 × 10^9 ^daily	Reduced SCORAD at the end of intervention and 16 weeks after intervention
	C = 28		SCORAD ≥ 25		Duration: 8 weeks	
Brouwer et al (2006)[36]	A1 = 16	< 5 months	Eczema and suspected cow's milk allergy	38%	A1: HF with LGG 5 × 10^9 ^cfu/100 mL of formula	No effect
	A2 = 13				A2: HF with *L. rhamnosus *5 × 10^9^/100 mL of formula	
	C = 13				Placebo: HF Duration: 12 weeks	
Sistek et al (2006)[39]	A = 29	1 to 10 years	All atopic (sensitized)	100%	*L. rhamnosus *and *B. lactis *2 × 10^10 ^daily	Reduced SCORAD in food-sensitized infants
	C = 30		SCORAD ≥ 10	(food, A = 66%, P = 80%)	Duration: 12 weeks	
Fölster-Holst et al (2006)[38]	A = 26	1 to 55 months	Moderate to severe	38%	LGG 10 × 10^9 ^daily	No effect
	C = 27				Duration: 8 weeks	
Grüber et al (2007)[37]	A = 54	3 to 12 months	Mild to moderate	55%	LGG capsules > 5 × 10^9 ^twice daily	No effect
	C = 48		SCORAD 15 to 40	(A = 62%, P = 47%)	Duration: 12 weeks	

A, active treatment group; C, placebo group.

Rosenfedlt et al [33] studied 43 children aged 1 to 13 years with eczema, in a double-blind, placebo-controlled crossover setting with a combination of 2 strains of bacteria (Table 1). A significantly greater proportion (56%) of patients experienced improvement after active treatment than after placebo (15%). A greater decrease in SCORAD index appeared among patients with atopic constitution after probiotic treatment than after placebo. *Lactobacillus fermentum *given for 2 months to 56 Australian 6- to 18-month-old infants, 71% of whom were sensitized, ameliorated their moderate-to-severe eczema [34].

Our own study entailed 230 infants in a randomized controlled trial where LGG, a mixture of 4 probiotics, or placebo was given for 1 month to infants with eczema. Half of them were diagnosed by a double-blind, placebo-controlled food challenge to have cow's milk allergy (CMA) [35]. Although probiotics had no additional therapeutic effect on healing of eczema in infants with or without CMA, in subgroup analysis, LGG compared to placebo was superior in infants with IgE-associated eczema (*P *= 0.027) [35]. The greatest effect of LGG was among patients with severe eczema (SCORAD > 30) and IgE positivity. Colonization of the supplemented probiotics was successful when analyzed from fecal samples.

No effect of probiotics was observed in 42 Dutch infants aged 1 to 5 months, given either LGG or another *L. rhamnosus *strain for 3 months in hydrolyzed formula compared to infants given hydrolyzed formula alone for eczema [36]. Two German studies also showed negative results: in a study on 102 infants, treatment with LGG for 3 months resulted in the same rate of improvement of eczema as that with placebo in the whole study group and in those with sensitization [37]. In the other study, 53 children with eczema, aged 1 to 55 months, were given either LGG or placebo for 2 months; no difference was observed in the clinical course of the groups [38]. Three months' treatment of young children (median age 4 years) with a combination of *L. rhamnosus *and *B. lactis *improved eczema in the subgroup sensitized to food, whereas in the whole study group there was no difference [39]. The effect was transient.

## Prevention of atopic disease

The first study on the possibility to prevent allergy in high-risk infants comprised 159 mothers from allergy-risk families who were randomized to receive LGG 4 weeks before delivery, and after delivery the breast-feeding mothers continued LGG but only bottle-fed infants received LGG directly (57%) until 6 months of age. At age 2, the prevalence of atopic eczema in the LGG group was 23% and in the placebo group 46%; the relative risk for developing eczema was significantly lower in the LGG group (Table 2) [40]. At age 4, 107 children came to a follow-up examination: in the LGG group eczema was diagnosed in 26% and in the placebo group 44%[41]; relative risk for eczema at age 4 remained significantly reduced (Table 2). An equal number of children in each group had respiratory allergic symptoms, and prevalence of sensitization was similar. Seventy-three percent of the children completed the 7-year follow-up. The prevalence of eczema remained significantly lower in the LGG group, 43% versus 66%; positive skin prick tests were detected in 32% of the children with no difference between the groups. The incidence of allergic airway diseases was low and similar in the study groups [42]. Kopp et al simulated the above study by giving LGG or placebo to 105 pregnant women carrying high-risk babies for 4 to 6 weeks before delivery and then to their infants for 6 months. They found a similar incidence of eczema (28% vs 27%) and of sensitization rates at age 2, but LGG was associated with an increased rate of recurrent wheezing episodes (26% vs 9%) [43].

**Table 2:** Randomized Placebo-Controlled Clinical Trials on Prevention of Eczema by Probiotics in High-Risk Infants

Study	No. Patients	Treatment Initiated	Follow-up (years)	Intervention and Amount of Probiotics (cfu) in the Active Group	Incidence of Eczema A/C	Effect on Eczema OR (95% CI)	Effect on IgE-Associated Eczema OR (95% CI)
Kalliomäki et al (2001)[40]	A = 77	Pregnant women and newborn babies	2	*L. rhamnosus *GG 1 × 10^10 ^2 to 4 weeks before delivery, 6 months after birth to lactating mothers, otherwise to bottle-fed infants	23% vs 46%	0.36	NA
	C = 82					(0.17-0.77)	--
Kalliomäki et al (2003)[41]	A = 53		4		26% vs 46%	0.42	--
	C = 54					(0.18-0.94)	--
Kalliomäki et al (2007)[42]	A = 53		7		43% vs 66%	0.58	--
	C = 62					(0.35-0.94)	--
Kukkonen et al (2007)[47]	A = 461	Pregnant women and newborn babies	2	*L. rhamnosus *GG 5 × 10^9^, *L. rhamnosus *LC705 5 × 10^9^, *B. breve *Bb99 2 × 10^8^, and *P. freudenreichii *ssp *shermanii *JS 2 × 10^9 ^plus prebiotic galacto-oligosaccharides from 36 gw daily for 6 months after birth	26% vs 32%	0.74	0.66
	C = 464					(0.55-0.98)	(0.46-0.95)
Kuitunen et al (2009)[48]	A = 445		5		39% vs 43%	0.85	--
	C = 446					(0.65-1.11)	--
Taylor et al (2007)[44]	High risk	Only newborn babies aged < 48 hours	1	*L. acidophilus *3 × 10^9 ^daily for 6 months after birth	43% vs 39%	1.18	2.18
	A = 89					(0.64-2.16)	(1.01-4.72)
	C = 88					--	--
Abrahamsson et al (2007)[45]	High risk	Pregnant women and newborn babies	2	*L. reuteri *1 × 10^8 ^from 36 gw daily to 12 months after birth	36% vs 34%	1.06	0.53
	A = 95					(0.58-1.93)	(0.24-1.16)
	C = 93					--	--
Kopp et al (2008)[43]	High risk	Pregnant women and newborn babies	2	*L. rhamnosus *GG 5 × 10^9 ^daily 4 to 6 weeks before delivery, 6 months after birth to lactating mothers, otherwise to bottle-fed infants	28% vs 27%	1.04	NA
	A = 50					(0.42-2.57)	--
	C = 44					--	--
Wickens et al (2008)[46]	High risk	Pregnant women and newborn babies	2	A1: *L. rhamnosus *HN001 6 × 10^9^ A2: *B. animalis *ssp *lactis *9 × 10^9 ^from 35 gw daily for 24 months after birth	15% vs 27%	0.51	0.51
	A1 = 144					(0.3-0.85)	(0.27-0.87)
	A2 = 152						
	C = 150						

A, active treatment group; C, control; cfu, colony-forming units.

In another randomized trial, *Lactobacillus acidophilus *(LAVRI-A1) or placebo was given only postnatally to 178 newborns of allergic women until 6 months of age [44]. At 1 year, no differences in the rates of eczema in the probiotic (43%) and placebo (39%) groups were found. Unexpectedly, increased frequency of sensitization was found in the probiotic group, 40%, compared to the placebo (24%) group. The proportion of children with atopic eczema having a positive skin prick test was greater in the probiotic group, 23/88 (26%), than in the placebo group, 12/86 (14%), *P *= 0.045.

Abrahamsson et al gave either *Lactobacillus reuteri *or placebo daily to pregnant mothers of high-risk families from 36 weeks of gestation until delivery and to their babies from birth until 1 year of age [45]. In the 188 infants at the 2-year follow-up, the cumulative incidence of eczema was 35% in both groups, but during the second year of life, atopic eczema was less common in the probiotic group (8%) than in the placebo group (20%). The cumulative incidence of sensitization as measured by serum antigen-specific IgE against egg white and cow's milk or in a skin prick test against egg, milk, cat, birch, or timothy tended to be lower in the probiotic group (18% vs 29%); when infants with maternal allergy were compared, the difference was significant (14% vs 31%, *P *= 0.02).

Wickens et al [46] randomized 512 mothers of high-risk infants to receive either *L. rhamnosus *HN001 or *Bifidobacterium animalis *subspecies or placebo from 35 weeks of gestation; to the infants the treatment was continued until age 2. *L. rhamnosus *significantly reduced risk of eczema compared with placebo, but the other strain bifidobacteria had no effect. IgE-associated eczema was reduced in the same way, but sensitization rates were similar by age 2.

We randomized for a double-blind placebo-controlled trial 1223 pregnant women carrying fetuses at increased risk for allergies [47]. These mothers used a mixture of 4 probiotic bacteria, or a placebo, from their 36th week of gestation. Their infants received the same probiotics plus prebiotic galacto-oligosaccharides for 6 months. In the probiotic group compared to the placebo group, fecal counts for all lactobacilli and bifidobacteria were significantly higher than those in the controls at age 6 months. Probiotic strains were also detected more frequently, recovery of the probiotics in the feces was transient, and no differences in the colonization patterns occurred at 2 years of age.

A total of 925 infants participated in the 2-year follow-up. The cumulative incidence of any allergic disease (food allergy, eczema, asthma, and allergic rhinitis) did not differ significantly between the probiotic (32%) and the placebo (35%) groups. However, probiotics compared to placebo tended to reduce all atopic (IgE-associated, assessed by skin prick test and or specific IgE > 0.7 kU/L)) diseases (Table 2). Eczema, which constituted 88% of all allergic diseases by age 2, occurred less frequently in the probiotic group (26%) than in the placebo group (32%). The preventive effect was more pronounced against atopic (IgE-associated) eczema, its incidence in the probiotic group (12%) was significantly lower than that in the placebo group (18%). Sensitization, however, was not affected.

At age 5, 891 (88%) of the group attended the follow-up examination [48]. The frequencies of allergic and IgE-associated allergic disease and sensitization in the probiotic and placebo groups were similar: 52.6% versus 54.9% and 29.5% versus 26.6% and 41.3% in both. There was also no difference in the frequencies of eczema (39.3% vs 43.3%), atopic eczema (24.0% vs 25.1%), allergic rhinitis (20.7% vs 19.1%), nor asthma (13.0% vs 14.1%) between the groups. However, caesarean-delivered children receiving probiotics were sensitized less frequently; the difference in prevalence of positive IgE antibodies to food allergens was significant, they had less IgE-associated allergic disease, and the cumulative prevalence for atopic eczema was significantly reduced (15.7% vs 30.4%) [48].

## Safety of probiotic treatment

No serious adverse effect has been reported in the studies using probiotics in infants and children to treat or prevent allergies. In our intervention, the early adverse symptoms that could be caused by probiotics (abdominal pains, excessive crying, and constipation) were equally common in the probiotic and placebo group [49]. The growth of the children in the groups was exactly the same. The concentrations of hemoglobin at ages 2 and 5 were the same, although at age 6 months those receiving probiotics had signs of iron deficiency [50].

Infants on probiotics in fact showed some favorable effects of the treatment. They had had less respiratory infection at age 6 to 24 months and had received less frequently antibiotics from birth to 6 month [49]. At age 6 months they more frequently had protective titers against Hemophilus influenzae B than those on placebo [51]. From a mean age of 7 months, 118 healthy infants consumed a formula supplemented with *B. lactis *and *Streptococcus thermophilus *for 7 months. It was well tolerated and safe and normal growth was observed [52].

## Mode of action of probiotics

A large number of studies describe immunologic effects of probiotics on human cells or on experimental animals. However, in our opinion the majority gives no information relevant to the human in vivo situation. Effects of probiotic bacteria on human cells do not reflect conditions in the intestine, where contact with bacteria takes place only for epithelial cells and for extensions of dendritic cells [53].

Majamaa and Isolauri inferred that probiotics reduce the inflammation in the intestine [30]. Inflammatory cytokine, tumor necrosis factor-*α *content was reduced in the fecal extracts of patients receiving LGG, whereas no change took place in the extracts from controls [30]. In a later study,[32] concentrations of urinary eosinophilic protein-x became lower in 2 groups receiving probiotic treatment and was unchanged in controls.

It has been suggested that probiotics act by reducing the permeability of the intestine [54]. In their double-blind placebocontrolled crossover study, probiotic treatment resulted in a lower ratio of lactulose/mannitol in the urine [54]. We however did not find any change in intestinal permeability during the treatment of infants with eczema with either LGG or a combination of probiotic strains [55].

We found no difference in the tumor necrosis factor-*α *content in the feces of patients receiving either probiotics or placebo [56]. However, LGG treatment resulted in a greater increase in concentration of IgA after a positive CM challenge test of IgE-mediated cow's milk allergic infants than that in controls [57]. In the prevention study, we discovered that high fecal IgA concentrations at age 6 months protected the infant/child from atopic (IgE-associated) diseases by age 2 years. Probiotics led to increased concentrations of inflammatory markers, fecal *α*1-antitrypsin and calprotectin, and tended to augment fecal IgA concentrations [58]. We therefore infer that in the intestine, probiotics may enhance both inflammation and immune defense of the gut.

When we studied the ability of peripheral blood mononuclear cells to secrete various cytokines before and after treatment with probiotics and placebo, we found the secretion of interferon-*γ *(IFN-*γ*) to be significantly lower in IgE-mediated cow's milk allergic infants than in infants without CMA [59]. Treatment with LGG resulted in a significant increase in the ability of peripheral blood mononuclear cells to secrete IFN-*γ *among patients with atopic eczema, the same group which benefited clinically from the treatment [59]. The same increase was observed for IFN-*γ *responses to mitogens and staphylococcal enterotoxin B in infants with eczema given *L. fermentum *VRI 003 [60]. Interestingly, in our study, the mixture of probiotics acted differently from LGG: Secretion of IL-4 increased significantly in infants with CMA during the intervention with the mixture, whereas LGG did not effect this cytokine secretion [59].

Both in the treatment and prevention study we found evidence that probiotics induce low-grade inflammation, which probably is associated with the healing/protective actions of probiotics. During treatment of eczema with LGG, we found a significant increase in blood concentration of C-reactive protein (CRP) in infants having had a favorable clinical effect, in those with IgE-associated eczema. The LGG treatment affected the serum concentration of IL-6, which was significantly increased in the group with increased CRP. IL-6 may thus induce the secretion of CRP in the liver. The effect of the mixture of probiotics differed; it had no affect on IL-6 levels, but was associated with a significant increase in IL-10 [56]. In infants with high risk of allergy, the mixture of probiotics was associated with an increase of CRP at age 6 months; they also had higher IL-10 levels. Furthermore, they had higher levels of serum IgA and IgE levels than those given placebo [61]. We, therefore, infer that probiotics induce a low-grade inflammation characterized as an increase in CRP, total IgA, total IgE, and IL-10 levels. These changes closely resemble those seen in helminth infections and are associated with induction of regulatory mechanisms and reduced incidence of allergy [62].

Commensal microbiota and their recognition by TLRs are important in host defense and directing specific immune responses of the gut and in development of food allergy in experimental animals [63,64]. Probiotic strains have the ability to adhere to gut epithelial cells, which may express TLRs [65] and stimulate these cells to produce cytokines. Extension of dendritic cells samples the intestinal lumen and functions in the development of immune responses in the gut [53]. These cells may be stimulated by probiotic bacteria. In vitro, isolated myeloid dendritic cells express TLR-2 and may be stimulated by LGG to express inflammatory cytokines [66]. We thus infer that stimulation of innate immunity may be the basis of the observed inflammatory signs and beneficial clinical effects.

## Conclusions

Studies with probiotics to treat and prevent allergy show promising, although highly variable, results as also discussed in 3 recent reviews [67-69]. Clearly, the major variable among the studies is the use of different bacterial strains; only results using the same strain and similar set-up are comparable.

We believe that the concept is valid: the intestine of a newborn and also an older infant may be transiently colonized with bacteria given orally. These bacteria have an effect on the immune system of the recipient and also have clinical effects. Probiotics have been effective in the treatment of eczema in infants, although the results are modest. In prevention, we saw the longest lasting results in the subgroup of children born by caesarean section. In that event we can introduce the probiotic to the intestine with low counts of bacteria and higher counts of given strains in the intestine may be reached. However, in all instances the colonization by given strains have been transient.

In attempts to prevent allergy in high-risk infants the results suggest that intervention should start with the mother during pregnancy to make sure that the birth canal of the mother is colonized by probiotics.

Whether both infants and their mothers should continue probiotics after birth is an open question; giving probiotics directly to infants is proven to result in colonization.

Finding the most efficient strain of probiotics is a big challenge. We do not believe in vitro studies can simulate conditions in vivo, although some qualities of probiotic bacteria may be found in those studies. Experimental animals have gut microbial flora, which for example in mice has less than 50% DNA identity with the human microbiota. Therefore, much caution is needed to apply results from experimental animal studies. Even in human experiments, we do not know what type of immune reaction should result from the ingestion of probiotics to prove their effect in allergy treatment and prevention. Furthermore, the immune response to probiotics may be genetically determined and differ in persons with and without allergy proneness.

For more efficient and long-lasting effects, we need more potent and longer lasting stimulation of the mucosal immune system. Maybe the intervention has to continue lifelong; its type has to be changed or added up at intervals. Challenges to find a safe and efficient intervention for the primary prevention of allergies are great, but first steps have been taken.
